# Acetonitrile­bis­(2,9-dimethyl-1,10-phen­an­throline)copper(II) bis­(tetra­fluorido­borate)

**DOI:** 10.1107/S1600536810042285

**Published:** 2010-10-23

**Authors:** Stephen P. Watton

**Affiliations:** a Department of Chemistry and Biochemistry, Central Connecticut State University, 1615 Stanley Street, New Britain, CT 06050, USA

## Abstract

The title compound, [Cu(CH_3_CN)(C_12_H_12_N_2_)_2_](BF_4_)_2_, crystallizes with two copper-containing cations and four tetra­fluoro­borate anions in the asymmetric unit. The structure represents a second crystal form of the salt, the first being an acetonitrile solvate [Watton (2009 ▶). *Acta Cryst.* E**65**, m585–m586]. The complex cation has a distorted trigonal-bipyramidal geometry, whereas the previous structure exhibits a distorted square-pyramidal geometry. One of the four BF_4_^−^ counter-ions is disordered, with a refined site occupancy of 0.8615 (17):0.1385 (17).

## Related literature

For the acetonitrile solvate structure, see: Watton (2009 ▶). For geometrical analysis, see: Addison *et al.* (1984 ▶); Holmes (1984 ▶); Watton (2010 ▶). For electrochemical behaviour of similar complexes, see: James & Williams (1961 ▶). For the characteristic colour of four-coordinate Cu(II) species, see: Miller *et al.* (1998 ▶).
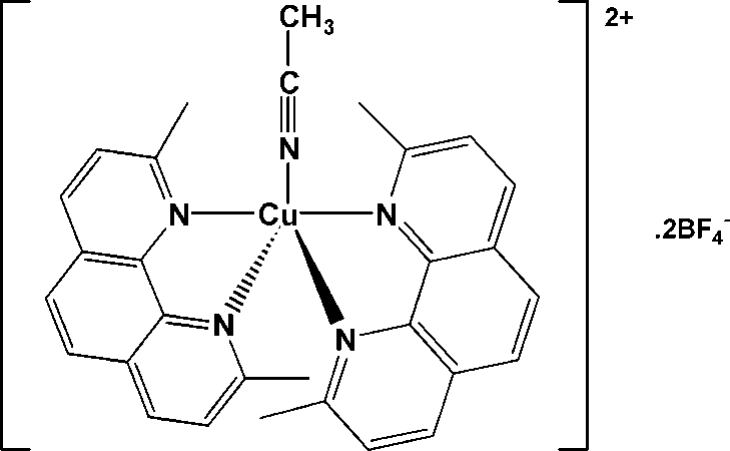


## Experimental

### 

#### Crystal data

[Cu(C_2_H_3_N)(C_12_H_12_N_2_)_2_](BF_4_)_2_
*M* *_r_* = 694.72
Monoclinic, 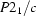

*a* = 14.7973 (3) Å
*b* = 18.5356 (3) Å
*c* = 22.5770 (4) Å
β = 105.2524 (18)°
*V* = 5974.23 (19) Å^3^
*Z* = 8
Mo *K*α radiation
μ = 0.81 mm^−1^
*T* = 293 K
0.20 × 0.20 × 0.15 mm

#### Data collection

Oxford Diffraction Sapphire 3 diffractometer
Absorption correction: multi-scan (*CrysAlis RED*; Oxford Diffraction, 2006 ▶) *T*_min_ = 0.765, *T*_max_ = 1.000
40609 measured reflections
19629 independent reflections
13249 reflections with *I* > 2σ(*I*)
*R*_int_ = 0.026

#### Refinement

*R*[*F* ^2^ > 2σ(*F* ^2^)] = 0.046
*wR*(*F* ^2^) = 0.122
*S* = 1.07
19629 reflections
855 parameters
30 restraints
H-atom parameters constrained
Δρ_max_ = 1.06 e Å^−3^
Δρ_min_ = −0.88 e Å^−3^

### 

Data collection: *CrysAlis CCD* (Oxford Diffraction, 2006 ▶); cell refinement: *CrysAlis RED* (Oxford Diffraction, 2006 ▶); data reduction: *CrysAlis RED*; program(s) used to solve structure: *SHELXS97* (Sheldrick, 2008 ▶); program(s) used to refine structure: *SHELXL97* (Sheldrick, 2008 ▶); molecular graphics: *ORTEP-3* (Farrugia, 1997 ▶); software used to prepare material for publication: *publCIF* (Westrip, 2010 ▶).

## Supplementary Material

Crystal structure: contains datablocks I, global. DOI: 10.1107/S1600536810042285/fj2351sup1.cif

Structure factors: contains datablocks I. DOI: 10.1107/S1600536810042285/fj2351Isup2.hkl

Additional supplementary materials:  crystallographic information; 3D view; checkCIF report

## Figures and Tables

**Fig. 1.**
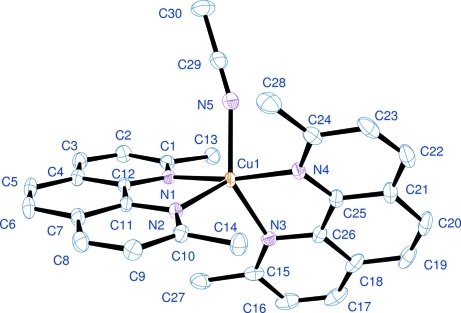
ORTEP of Cation 1 showing atom numbering scheme. Hydrogen atoms omitted for clarity and thermal ellipsoids drawn at 50% probability level.

**Fig. 2.**
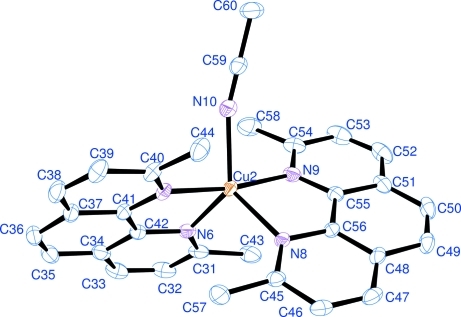
ORTEP of Cation 2 showing atom numbering scheme. Hydrogen atoms omitted for clarity and thermal ellipsoids drawn at 50% probability level.

## supplementary crystallographic information

### Comment

The structure was obtained as part of a study of how substituents at the 2- and 9- positions of the phenanthroline ligand affect the behavior of the copper complexes. The crystal was obtained during an attempt to prepare a larger amount of the previously reported complex (Watton, 2009), which differs from the current structure in that it contains two molecules of acetonitrile per cation in the crystal lattice, while the current form is unsolvated. The appearance of this new crystal form was unexpected, and it is not fully understood how the preparative conditions affect the particular crystal form that is obtained. This aspect of the chemistry is currently under study.

The two crystal forms of the cation differ significantly in their structures. There is substantial distortion from idealized geometry in both cases, as would be expected from the small bite-angle of the phen ligand. The previous compund is best described as having a distorted square pyramidal geometry at copper; the τ descriptor of Addison *et al.* (Addison, 1984), has a value of 0.24 (where τ = 0 for ideal square planar geometry and τ = 1 for trigonal bipyramidal),and the analysis of Holmes (Holmes, 1984) indicates that the structure is 73% along the Berry pseudorotation coordinate (D3h —> C2v —> C4v). By contrast, the current stucture is much closer to tbp (τ =. 63 and 0.72 for the two cations, 34.8% and 27.8% along pseudorotation coordinate). It is noted that a less sterically demanding ligand, 2-methylphenanthroline, affords a structure that is essentially tbp (τ = 0.9, 8.2%)(Watton, 2010). The distortions from idealized geometry in both crystal forms of [Cu(2,9-DMP)_2_]^2+^ are consistent with the observation that the 2,9-dimethyl substituents destabilize the 5-coordinate cupric form of the bis-phenanthroline complex with respect to the less sterically hindered 4-coordinate cuprous form, as manifested in the more favorable reduction potential of the dimethyl complex with respect to the unsubstituted analog (James, 1961). The steric strain results in quite different distortions within the two structures, however. Whereas the solvated structure exhibits substantial bowing of the phen ligands from the ideal planar geometry of an aromatic polycyclic ligand, no such bowing is observed in the present structure. In both cases, the copper ions lie out of the plane of the phenanthroline ligands, but the average deviation of the copper ions from the least-squares planes of the ligands is significantly greater (average = 0.55 (18) A) in the previous structure than it is in the current one (average = 0.29 (1) A). Apparently to offset these lesser distortions in the current structure, there is a significant deviation of the coordinated acetonitrile ligand from the expected linear geometry (Cu—N—C = 163.5°); the solvated structure showed far less distortion (Cu—N—C = 173.4 °). Interestingly, this apparent destabilization of the Cu-acetonitrile bond results in a difference in chemical properties for the two crystal forms: While the solvated crystals are stable for extended periods of time when removed from the mother liquor, the unsolvated crystals undergo what appears to be a rapid deliquescence, which is accompanied by a change in color from green to purple. Previous studies (Miller, 1998) have shown this color to be characteristic of the unusual four-coordinate Cu(II) species. Further study of this interesting behavior is in progress.

### Crystal data

**Table:**

[Cu(C_2_H_3_N)(C_12_H_12_N_2_)_2_](BF_4_)_2_	*F*(000) = 2824
*M**_r_* = 694.72	*D*_x_ = 1.545 Mg m^−^^3^
Monoclinic, *P*2_1_/*c*	Melting point: 573 K
Hall symbol: -P 2ybc	Mo *K*α radiation, λ = 0.71073 Å
*a* = 14.7973 (3) Å	Cell parameters from 19467 reflections
*b* = 18.5356 (3) Å	θ = 4.3–32.6°
*c* = 22.5770 (4) Å	µ = 0.81 mm^−^^1^
β = 105.2524 (18)°	*T* = 293 K
*V* = 5974.23 (19) Å^3^	Block, green
*Z* = 8	0.20 × 0.20 × 0.15 mm

### Data collection

**Table:**

Oxford Diffraction Sapphire 3 diffractometer	19629 independent reflections
Radiation source: fine-focus sealed tube	13249 reflections with *I* > 2σ(*I*)
graphite	*R*_int_ = 0.026
ω scans	θ_max_ = 32.7°, θ_min_ = 4.3°
Absorption correction: multi-scan (*CrysAlis RED*; Oxford Diffraction, 2006)	*h* = −21→19
*T*_min_ = 0.765, *T*_max_ = 1.000	*k* = −22→28
40609 measured reflections	*l* = −31→34

### Refinement

**Table:**

Refinement on *F*^2^	Primary atom site location: structure-invariant direct methods
Least-squares matrix: full	Secondary atom site location: difference Fourier map
*R*[*F*^2^ > 2σ(*F*^2^)] = 0.046	Hydrogen site location: inferred from neighbouring sites
*wR*(*F*^2^) = 0.122	H-atom parameters constrained
*S* = 1.07	*w* = 1/[σ^2^(*F*_o_^2^) + (0.0659*P*)^2^ + 0.084*P*] where *P* = (*F*_o_^2^ + 2*F*_c_^2^)/3
19629 reflections	(Δ/σ)_max_ = 0.002
855 parameters	Δρ_max_ = 1.06 e Å^−^^3^
30 restraints	Δρ_min_ = −0.88 e Å^−^^3^

### Special details

**Table:**

Geometry. All e.s.d.'s (except the e.s.d. in the dihedral angle between two l.s. planes) are estimated using the full covariance matrix. The cell e.s.d.'s are taken into account individually in the estimation of e.s.d.'s in distances, angles and torsion angles; correlations between e.s.d.'s in cell parameters are only used when they are defined by crystal symmetry. An approximate (isotropic) treatment of cell e.s.d.'s is used for estimating e.s.d.'s involving l.s. planes.
Refinement. Refinement of *F*^2^ against ALL reflections. The weighted *R*-factor *wR* and goodness of fit *S* are based on *F*^2^, conventional *R*-factors *R* are based on *F*, with *F* set to zero for negative *F*^2^. The threshold expression of *F*^2^ > σ(*F*^2^) is used only for calculating *R*-factors(gt) *etc*. and is not relevant to the choice of reflections for refinement. *R*-factors based on *F*^2^ are statistically about twice as large as those based on *F*, and *R*- factors based on ALL data will be even larger.

### Fractional atomic coordinates and isotropic or equivalent isotropic displacement parameters (Å^2^)

**Table:**

	*x*	*y*	*z*	*U*_iso_*/*U*_eq_	Occ. (<1)
Cu1	0.084941 (15)	0.276621 (12)	0.343942 (10)	0.01537 (6)	
N1	−0.00314 (10)	0.21950 (8)	0.27929 (7)	0.0154 (3)	
N2	−0.03933 (11)	0.33834 (8)	0.33783 (7)	0.0162 (3)	
N3	0.10759 (11)	0.20701 (8)	0.41990 (7)	0.0171 (3)	
N4	0.18867 (11)	0.33074 (8)	0.40062 (7)	0.0169 (3)	
N5	0.15524 (13)	0.29072 (10)	0.27572 (8)	0.0279 (4)	
C1	0.01772 (13)	0.15984 (10)	0.25236 (8)	0.0183 (4)	
C2	−0.04581 (14)	0.13204 (11)	0.19916 (9)	0.0219 (4)	
H2	−0.0298	0.0913	0.1801	0.026*	
C3	−0.13060 (14)	0.16454 (11)	0.17550 (9)	0.0217 (4)	
H3	−0.1723	0.1458	0.1406	0.026*	
C4	−0.15453 (13)	0.22663 (10)	0.20424 (8)	0.0182 (3)	
C5	−0.24262 (14)	0.26302 (11)	0.18386 (9)	0.0229 (4)	
H5	−0.2866	0.2465	0.1491	0.028*	
C6	−0.26277 (14)	0.32106 (11)	0.21441 (9)	0.0235 (4)	
H6	−0.3205	0.3438	0.2004	0.028*	
C7	−0.19627 (13)	0.34786 (10)	0.26804 (8)	0.0187 (4)	
C8	−0.21440 (14)	0.40614 (10)	0.30333 (9)	0.0215 (4)	
H8	−0.2723	0.4291	0.2925	0.026*	
C9	−0.14640 (14)	0.42887 (10)	0.35361 (9)	0.0214 (4)	
H9	−0.1581	0.4676	0.3767	0.026*	
C10	−0.05858 (13)	0.39375 (10)	0.37051 (8)	0.0180 (3)	
C11	−0.10760 (12)	0.31515 (9)	0.28789 (8)	0.0154 (3)	
C12	−0.08763 (12)	0.25267 (10)	0.25616 (8)	0.0151 (3)	
C13	0.10864 (14)	0.12258 (11)	0.27908 (9)	0.0222 (4)	
H13A	0.1407	0.1458	0.3168	0.033*	
H13B	0.1467	0.1249	0.2506	0.033*	
H13C	0.0971	0.0730	0.2871	0.033*	
C14	0.01427 (14)	0.41765 (11)	0.42699 (9)	0.0239 (4)	
H14A	0.0429	0.3761	0.4497	0.036*	
H14B	−0.0148	0.4464	0.4522	0.036*	
H14C	0.0612	0.4457	0.4152	0.036*	
C15	0.06095 (15)	0.14918 (11)	0.43189 (9)	0.0229 (4)	
C16	0.09939 (18)	0.10427 (12)	0.48283 (10)	0.0308 (5)	
H16	0.0668	0.0636	0.4896	0.037*	
C17	0.18394 (18)	0.12024 (12)	0.52207 (9)	0.0320 (5)	
H17	0.2095	0.0900	0.5551	0.038*	
C18	0.23232 (15)	0.18202 (11)	0.51279 (9)	0.0248 (4)	
C19	0.31869 (16)	0.20535 (13)	0.55351 (9)	0.0303 (5)	
H19	0.3467	0.1775	0.5877	0.036*	
C20	0.35999 (15)	0.26690 (13)	0.54312 (9)	0.0311 (5)	
H20	0.4167	0.2804	0.5699	0.037*	
C21	0.31817 (13)	0.31238 (11)	0.49134 (9)	0.0237 (4)	
C22	0.35496 (14)	0.37909 (13)	0.48009 (10)	0.0293 (5)	
H22	0.4111	0.3955	0.5057	0.035*	
C23	0.30806 (15)	0.41974 (12)	0.43140 (11)	0.0290 (5)	
H23	0.3319	0.4645	0.4246	0.035*	
C24	0.22373 (14)	0.39501 (11)	0.39107 (9)	0.0228 (4)	
C25	0.23381 (13)	0.29031 (10)	0.45008 (8)	0.0182 (4)	
C26	0.19076 (13)	0.22427 (10)	0.46050 (8)	0.0184 (4)	
C27	−0.03382 (16)	0.13256 (12)	0.39053 (10)	0.0299 (5)	
H27A	−0.0669	0.1768	0.3776	0.045*	
H27B	−0.0684	0.1037	0.4123	0.045*	
H27C	−0.0268	0.1065	0.3552	0.045*	
C28	0.17413 (17)	0.43980 (12)	0.33721 (11)	0.0323 (5)	
H28A	0.2048	0.4347	0.3049	0.048*	
H28B	0.1754	0.4895	0.3493	0.048*	
H28C	0.1103	0.4239	0.3229	0.048*	
C29	0.17157 (15)	0.30088 (12)	0.22990 (10)	0.0259 (4)	
C30	0.19169 (18)	0.31546 (13)	0.17158 (10)	0.0340 (5)	
H30A	0.1536	0.3549	0.1516	0.051*	
H30B	0.1781	0.2733	0.1460	0.051*	
H30C	0.2566	0.3278	0.1785	0.051*	
Cu2	0.389985 (16)	0.691709 (13)	0.105797 (10)	0.01934 (6)	
N6	0.40860 (12)	0.80441 (10)	0.10026 (7)	0.0220 (3)	
N7	0.27073 (11)	0.71383 (9)	0.04267 (7)	0.0196 (3)	
N8	0.47331 (11)	0.63926 (9)	0.05485 (7)	0.0179 (3)	
N9	0.49699 (12)	0.66233 (9)	0.17556 (7)	0.0220 (3)	
N10	0.30781 (12)	0.63636 (10)	0.15327 (9)	0.0301 (4)	
C31	0.47989 (15)	0.84849 (12)	0.12578 (9)	0.0258 (4)	
C32	0.46991 (17)	0.92389 (13)	0.12152 (10)	0.0329 (5)	
H32	0.5195	0.9534	0.1414	0.040*	
C33	0.38771 (17)	0.95422 (12)	0.08832 (10)	0.0312 (5)	
H33	0.3813	1.0041	0.0858	0.037*	
C34	0.31299 (16)	0.90937 (11)	0.05798 (9)	0.0250 (4)	
C35	0.22656 (17)	0.93593 (12)	0.01946 (10)	0.0291 (5)	
H35	0.2177	0.9854	0.0141	0.035*	
C36	0.15750 (16)	0.89042 (12)	−0.00933 (9)	0.0281 (4)	
H36	0.1019	0.9090	−0.0340	0.034*	
C37	0.16900 (14)	0.81373 (11)	−0.00228 (9)	0.0237 (4)	
C38	0.10079 (15)	0.76359 (13)	−0.03216 (10)	0.0291 (5)	
H38	0.0435	0.7795	−0.0567	0.035*	
C39	0.11933 (15)	0.69138 (12)	−0.02494 (10)	0.0281 (4)	
H39	0.0747	0.6581	−0.0450	0.034*	
C40	0.20568 (14)	0.66717 (11)	0.01275 (9)	0.0231 (4)	
C41	0.25367 (14)	0.78642 (10)	0.03519 (9)	0.0201 (4)	
C42	0.32663 (14)	0.83454 (11)	0.06588 (9)	0.0209 (4)	
C43	0.57260 (16)	0.81640 (14)	0.15865 (11)	0.0346 (5)	
H43A	0.5715	0.8030	0.1995	0.052*	
H43B	0.6213	0.8512	0.1606	0.052*	
H43C	0.5844	0.7744	0.1369	0.052*	
C44	0.22558 (15)	0.58754 (11)	0.01864 (11)	0.0288 (5)	
H44A	0.2917	0.5800	0.0348	0.043*	
H44B	0.2050	0.5652	−0.0210	0.043*	
H44C	0.1928	0.5666	0.0459	0.043*	
C45	0.46565 (14)	0.63376 (11)	−0.00539 (9)	0.0214 (4)	
C46	0.52727 (15)	0.59019 (11)	−0.02811 (9)	0.0244 (4)	
H46	0.5192	0.5858	−0.0702	0.029*	
C47	0.59841 (15)	0.55459 (11)	0.01151 (10)	0.0258 (4)	
H47	0.6391	0.5261	−0.0035	0.031*	
C48	0.61049 (13)	0.56075 (10)	0.07503 (10)	0.0217 (4)	
C49	0.68461 (14)	0.52621 (11)	0.11996 (11)	0.0281 (5)	
H49	0.7267	0.4967	0.1072	0.034*	
C50	0.69392 (14)	0.53596 (11)	0.18022 (11)	0.0300 (5)	
H50	0.7417	0.5121	0.2085	0.036*	
C51	0.63208 (14)	0.58223 (11)	0.20202 (10)	0.0251 (4)	
C52	0.64206 (16)	0.59802 (12)	0.26422 (10)	0.0325 (5)	
H52	0.6896	0.5765	0.2943	0.039*	
C53	0.58196 (18)	0.64482 (13)	0.28023 (10)	0.0344 (5)	
H53	0.5893	0.6557	0.3214	0.041*	
C54	0.50824 (16)	0.67732 (12)	0.23509 (9)	0.0279 (5)	
C55	0.55759 (13)	0.61575 (10)	0.15911 (9)	0.0195 (4)	
C56	0.54592 (13)	0.60427 (10)	0.09476 (9)	0.0179 (3)	
C57	0.39157 (16)	0.67533 (13)	−0.05023 (9)	0.0297 (5)	
H57A	0.3345	0.6479	−0.0606	0.045*	
H57B	0.4116	0.6844	−0.0867	0.045*	
H57C	0.3810	0.7204	−0.0322	0.045*	
C58	0.44347 (19)	0.72961 (14)	0.25291 (11)	0.0375 (6)	
H58A	0.4054	0.7525	0.2168	0.056*	
H58B	0.4793	0.7655	0.2797	0.056*	
H58C	0.4040	0.7045	0.2737	0.056*	
C59	0.27828 (14)	0.59525 (12)	0.18046 (10)	0.0264 (4)	
C60	0.24070 (17)	0.54244 (13)	0.21520 (11)	0.0338 (5)	
H60A	0.1794	0.5279	0.1919	0.051*	
H60B	0.2367	0.5634	0.2533	0.051*	
H60C	0.2812	0.5011	0.2234	0.051*	
B1	0.14194 (16)	0.37274 (12)	0.59292 (10)	0.0216 (4)	
F1	0.12278 (9)	0.31320 (6)	0.55422 (6)	0.0299 (3)	
F2	0.15876 (12)	0.35016 (8)	0.65311 (6)	0.0456 (4)	
F3	0.06508 (11)	0.41852 (8)	0.58040 (8)	0.0512 (4)	
F4	0.22077 (11)	0.40904 (8)	0.58686 (8)	0.0469 (4)	
B2	0.39281 (17)	0.60964 (13)	0.40230 (11)	0.0255 (5)	
F5	0.33071 (12)	0.57495 (9)	0.35454 (8)	0.0605 (5)	
F6	0.44297 (10)	0.55965 (8)	0.44397 (8)	0.0523 (5)	
F7	0.33939 (13)	0.65099 (9)	0.43110 (7)	0.0565 (5)	
F8	0.44967 (13)	0.65558 (14)	0.38094 (8)	0.0811 (7)	
B3	0.98516 (18)	0.44247 (14)	0.15311 (12)	0.0312 (5)	
F9	1.02798 (10)	0.48400 (8)	0.20389 (6)	0.0405 (3)	
F10	0.98237 (12)	0.37088 (8)	0.16878 (8)	0.0501 (4)	
F11	0.89527 (11)	0.46847 (9)	0.12734 (8)	0.0576 (5)	
F12	1.03623 (13)	0.44934 (8)	0.10927 (7)	0.0522 (4)	
B4	0.4382 (2)	0.44043 (18)	0.18722 (13)	0.0285 (7)	0.8615 (17)
F13	0.52285 (13)	0.41538 (11)	0.17988 (8)	0.0520 (5)	0.8615 (17)
F14	0.45417 (14)	0.49308 (11)	0.23150 (9)	0.0548 (5)	0.8615 (17)
F15	0.39075 (18)	0.38431 (14)	0.20358 (14)	0.0916 (10)	0.8615 (17)
F16	0.38341 (16)	0.46846 (12)	0.13232 (8)	0.0597 (6)	0.8615 (17)
B4A	0.4575 (11)	0.4282 (10)	0.2016 (7)	0.0285 (7)	0.1385 (17)
F13A	0.5483 (7)	0.4392 (7)	0.2375 (5)	0.0520 (5)	0.1385 (17)
F14A	0.4001 (8)	0.4306 (7)	0.2415 (5)	0.0548 (5)	0.1385 (17)
F15A	0.4589 (11)	0.3639 (7)	0.1725 (7)	0.0916 (10)	0.1385 (17)
F16A	0.4292 (10)	0.4821 (8)	0.1576 (5)	0.0597 (6)	0.1385 (17)

### Atomic displacement parameters (Å^2^)

**Table:**

	*U*^11^	*U*^22^	*U*^33^	*U*^12^	*U*^13^	*U*^23^
Cu1	0.01123 (10)	0.01830 (11)	0.01463 (10)	−0.00064 (8)	−0.00004 (7)	0.00094 (8)
N1	0.0135 (7)	0.0177 (7)	0.0147 (6)	0.0018 (6)	0.0031 (5)	0.0010 (6)
N2	0.0138 (7)	0.0181 (7)	0.0157 (7)	−0.0008 (6)	0.0020 (5)	0.0006 (6)
N3	0.0175 (7)	0.0187 (7)	0.0154 (7)	0.0014 (6)	0.0051 (6)	0.0006 (6)
N4	0.0131 (7)	0.0185 (7)	0.0180 (7)	−0.0015 (6)	0.0025 (6)	−0.0015 (6)
N5	0.0222 (9)	0.0385 (10)	0.0216 (8)	−0.0094 (7)	0.0032 (7)	−0.0013 (7)
C1	0.0174 (9)	0.0192 (9)	0.0190 (8)	−0.0002 (7)	0.0059 (7)	0.0015 (7)
C2	0.0228 (10)	0.0211 (9)	0.0216 (9)	0.0002 (8)	0.0055 (7)	−0.0038 (7)
C3	0.0211 (9)	0.0232 (9)	0.0185 (8)	−0.0040 (8)	0.0010 (7)	−0.0031 (7)
C4	0.0171 (8)	0.0190 (9)	0.0161 (8)	−0.0003 (7)	0.0000 (6)	−0.0002 (7)
C5	0.0179 (9)	0.0242 (10)	0.0209 (9)	−0.0012 (7)	−0.0052 (7)	0.0003 (7)
C6	0.0148 (9)	0.0249 (10)	0.0253 (9)	0.0033 (7)	−0.0043 (7)	0.0021 (8)
C7	0.0145 (8)	0.0187 (9)	0.0210 (8)	0.0019 (7)	0.0014 (7)	0.0032 (7)
C8	0.0171 (9)	0.0198 (9)	0.0261 (9)	0.0052 (7)	0.0032 (7)	0.0024 (8)
C9	0.0231 (9)	0.0183 (9)	0.0226 (9)	0.0044 (8)	0.0055 (7)	−0.0014 (7)
C10	0.0184 (9)	0.0172 (8)	0.0173 (8)	0.0003 (7)	0.0027 (7)	0.0010 (7)
C11	0.0133 (8)	0.0167 (8)	0.0148 (7)	−0.0001 (6)	0.0009 (6)	0.0017 (6)
C12	0.0135 (8)	0.0157 (8)	0.0149 (7)	0.0000 (6)	0.0015 (6)	0.0018 (6)
C13	0.0187 (9)	0.0243 (10)	0.0241 (9)	0.0067 (8)	0.0063 (7)	0.0003 (8)
C14	0.0226 (10)	0.0245 (10)	0.0220 (9)	0.0023 (8)	0.0010 (7)	−0.0059 (8)
C15	0.0299 (11)	0.0206 (9)	0.0220 (9)	−0.0009 (8)	0.0135 (8)	−0.0007 (7)
C16	0.0479 (14)	0.0213 (10)	0.0282 (10)	0.0020 (10)	0.0190 (10)	0.0039 (8)
C17	0.0522 (15)	0.0266 (11)	0.0191 (9)	0.0173 (10)	0.0125 (9)	0.0055 (8)
C18	0.0311 (11)	0.0277 (10)	0.0145 (8)	0.0160 (9)	0.0044 (7)	−0.0007 (7)
C19	0.0315 (11)	0.0378 (12)	0.0171 (9)	0.0219 (10)	−0.0016 (8)	−0.0026 (8)
C20	0.0185 (10)	0.0506 (14)	0.0188 (9)	0.0160 (9)	−0.0045 (7)	−0.0125 (9)
C21	0.0145 (9)	0.0343 (11)	0.0208 (9)	0.0048 (8)	0.0018 (7)	−0.0129 (8)
C22	0.0152 (9)	0.0424 (13)	0.0299 (10)	−0.0038 (9)	0.0049 (8)	−0.0180 (10)
C23	0.0230 (10)	0.0279 (11)	0.0398 (12)	−0.0114 (9)	0.0147 (9)	−0.0133 (9)
C24	0.0197 (9)	0.0236 (9)	0.0274 (10)	−0.0028 (8)	0.0102 (8)	−0.0038 (8)
C25	0.0144 (8)	0.0228 (9)	0.0167 (8)	0.0045 (7)	0.0027 (6)	−0.0066 (7)
C26	0.0181 (9)	0.0205 (9)	0.0155 (8)	0.0074 (7)	0.0025 (6)	−0.0022 (7)
C27	0.0331 (12)	0.0303 (11)	0.0296 (10)	−0.0131 (9)	0.0144 (9)	−0.0023 (9)
C28	0.0337 (12)	0.0231 (10)	0.0419 (13)	−0.0039 (9)	0.0132 (10)	0.0066 (9)
C29	0.0227 (10)	0.0299 (10)	0.0244 (9)	−0.0084 (8)	0.0052 (8)	−0.0034 (8)
C30	0.0423 (14)	0.0395 (13)	0.0254 (10)	−0.0071 (11)	0.0180 (10)	−0.0009 (9)
Cu2	0.01406 (11)	0.02553 (13)	0.01898 (11)	0.00282 (9)	0.00535 (8)	0.00247 (9)
N6	0.0189 (8)	0.0301 (9)	0.0178 (7)	−0.0011 (7)	0.0060 (6)	0.0043 (7)
N7	0.0153 (7)	0.0230 (8)	0.0218 (7)	0.0026 (6)	0.0075 (6)	−0.0013 (6)
N8	0.0126 (7)	0.0236 (8)	0.0174 (7)	0.0002 (6)	0.0038 (6)	0.0034 (6)
N9	0.0208 (8)	0.0271 (8)	0.0179 (7)	−0.0022 (7)	0.0046 (6)	0.0041 (7)
N10	0.0184 (8)	0.0386 (10)	0.0336 (10)	0.0034 (8)	0.0074 (7)	0.0125 (8)
C31	0.0241 (10)	0.0348 (11)	0.0190 (9)	−0.0086 (9)	0.0065 (8)	0.0044 (8)
C32	0.0379 (13)	0.0366 (12)	0.0248 (10)	−0.0145 (10)	0.0090 (9)	0.0012 (9)
C33	0.0428 (13)	0.0244 (10)	0.0286 (10)	−0.0071 (10)	0.0134 (10)	0.0024 (9)
C34	0.0315 (11)	0.0248 (10)	0.0213 (9)	0.0007 (8)	0.0119 (8)	0.0035 (8)
C35	0.0360 (12)	0.0264 (10)	0.0273 (10)	0.0088 (9)	0.0122 (9)	0.0078 (9)
C36	0.0277 (11)	0.0328 (11)	0.0243 (9)	0.0118 (9)	0.0077 (8)	0.0073 (9)
C37	0.0202 (9)	0.0310 (11)	0.0206 (9)	0.0074 (8)	0.0064 (7)	0.0032 (8)
C38	0.0173 (9)	0.0431 (13)	0.0251 (10)	0.0065 (9)	0.0024 (8)	0.0010 (9)
C39	0.0175 (9)	0.0361 (12)	0.0294 (10)	0.0003 (9)	0.0037 (8)	−0.0063 (9)
C40	0.0169 (9)	0.0265 (10)	0.0277 (10)	0.0010 (8)	0.0091 (7)	−0.0040 (8)
C41	0.0182 (9)	0.0231 (9)	0.0204 (8)	0.0037 (7)	0.0077 (7)	0.0013 (7)
C42	0.0207 (9)	0.0255 (9)	0.0177 (8)	0.0013 (8)	0.0072 (7)	0.0027 (7)
C43	0.0228 (11)	0.0468 (14)	0.0307 (11)	−0.0119 (10)	0.0008 (9)	0.0114 (10)
C44	0.0233 (10)	0.0240 (10)	0.0401 (12)	0.0014 (8)	0.0105 (9)	−0.0064 (9)
C45	0.0177 (9)	0.0257 (10)	0.0214 (9)	−0.0038 (7)	0.0064 (7)	0.0022 (8)
C46	0.0281 (11)	0.0238 (10)	0.0256 (9)	−0.0064 (8)	0.0149 (8)	−0.0022 (8)
C47	0.0244 (10)	0.0202 (9)	0.0389 (11)	−0.0034 (8)	0.0192 (9)	−0.0026 (9)
C48	0.0131 (8)	0.0173 (9)	0.0360 (11)	−0.0020 (7)	0.0090 (8)	0.0017 (8)
C49	0.0139 (9)	0.0216 (10)	0.0482 (13)	0.0013 (7)	0.0069 (9)	0.0050 (9)
C50	0.0128 (9)	0.0232 (10)	0.0478 (13)	−0.0012 (7)	−0.0029 (8)	0.0110 (9)
C51	0.0166 (9)	0.0235 (10)	0.0305 (10)	−0.0066 (8)	−0.0021 (8)	0.0083 (8)
C52	0.0300 (12)	0.0310 (11)	0.0270 (10)	−0.0095 (9)	−0.0093 (9)	0.0090 (9)
C53	0.0420 (14)	0.0385 (13)	0.0176 (9)	−0.0142 (11)	−0.0012 (9)	0.0019 (9)
C54	0.0333 (12)	0.0307 (11)	0.0203 (9)	−0.0087 (9)	0.0082 (8)	0.0011 (8)
C55	0.0133 (8)	0.0227 (9)	0.0208 (8)	−0.0042 (7)	0.0013 (7)	0.0039 (7)
C56	0.0115 (8)	0.0193 (8)	0.0228 (8)	−0.0024 (7)	0.0043 (7)	0.0023 (7)
C57	0.0268 (11)	0.0416 (13)	0.0195 (9)	0.0035 (9)	0.0042 (8)	0.0065 (9)
C58	0.0497 (15)	0.0414 (13)	0.0269 (11)	−0.0018 (12)	0.0201 (11)	−0.0033 (10)
C59	0.0161 (9)	0.0357 (11)	0.0272 (10)	0.0022 (8)	0.0051 (8)	0.0062 (9)
C60	0.0294 (12)	0.0408 (13)	0.0340 (11)	−0.0063 (10)	0.0132 (9)	0.0082 (10)
B1	0.0192 (10)	0.0226 (10)	0.0212 (10)	0.0004 (8)	0.0023 (8)	−0.0028 (8)
F1	0.0321 (7)	0.0260 (6)	0.0290 (6)	0.0022 (5)	0.0038 (5)	−0.0085 (5)
F2	0.0631 (10)	0.0495 (9)	0.0227 (6)	−0.0056 (8)	0.0088 (7)	0.0003 (6)
F3	0.0365 (8)	0.0397 (8)	0.0613 (10)	0.0185 (7)	−0.0156 (7)	−0.0226 (7)
F4	0.0479 (9)	0.0371 (8)	0.0653 (10)	−0.0168 (7)	0.0318 (8)	−0.0054 (7)
B2	0.0199 (11)	0.0285 (12)	0.0270 (11)	−0.0070 (9)	0.0044 (9)	−0.0062 (9)
F5	0.0423 (9)	0.0589 (10)	0.0617 (11)	−0.0101 (8)	−0.0191 (8)	−0.0202 (9)
F6	0.0285 (8)	0.0289 (7)	0.0799 (12)	0.0035 (6)	−0.0205 (8)	−0.0045 (7)
F7	0.0745 (12)	0.0604 (10)	0.0370 (8)	0.0303 (9)	0.0188 (8)	0.0061 (8)
F8	0.0508 (11)	0.1460 (19)	0.0461 (10)	−0.0541 (12)	0.0120 (8)	0.0185 (11)
B3	0.0258 (12)	0.0322 (13)	0.0323 (12)	0.0063 (10)	0.0019 (10)	−0.0033 (11)
F9	0.0326 (8)	0.0498 (8)	0.0341 (7)	−0.0024 (6)	0.0000 (6)	−0.0082 (6)
F10	0.0499 (10)	0.0353 (8)	0.0658 (11)	−0.0008 (7)	0.0165 (8)	0.0064 (8)
F11	0.0312 (8)	0.0655 (11)	0.0608 (10)	0.0144 (8)	−0.0152 (7)	−0.0131 (9)
F12	0.0736 (12)	0.0443 (9)	0.0484 (9)	0.0118 (8)	0.0331 (9)	0.0023 (7)
B4	0.0280 (16)	0.0347 (17)	0.0206 (15)	−0.0020 (13)	0.0024 (12)	−0.0024 (13)
F13	0.0415 (11)	0.0712 (13)	0.0447 (10)	0.0222 (10)	0.0139 (8)	0.0047 (9)
F14	0.0473 (11)	0.0650 (12)	0.0449 (10)	0.0035 (9)	−0.0004 (9)	−0.0260 (9)
F15	0.0612 (16)	0.0842 (17)	0.120 (2)	−0.0425 (14)	0.0068 (15)	0.0368 (17)
F16	0.0703 (15)	0.0779 (14)	0.0236 (9)	0.0466 (12)	−0.0008 (9)	−0.0059 (9)
B4A	0.0280 (16)	0.0347 (17)	0.0206 (15)	−0.0020 (13)	0.0024 (12)	−0.0024 (13)
F13A	0.0415 (11)	0.0712 (13)	0.0447 (10)	0.0222 (10)	0.0139 (8)	0.0047 (9)
F14A	0.0473 (11)	0.0650 (12)	0.0449 (10)	0.0035 (9)	−0.0004 (9)	−0.0260 (9)
F15A	0.0612 (16)	0.0842 (17)	0.120 (2)	−0.0425 (14)	0.0068 (15)	0.0368 (17)
F16A	0.0703 (15)	0.0779 (14)	0.0236 (9)	0.0466 (12)	−0.0008 (9)	−0.0059 (9)

### Geometric parameters (Å, °)

**Table:**

Cu1—N1	1.9872 (15)	N8—C56	1.370 (2)
Cu1—N4	1.9915 (15)	N9—C54	1.339 (3)
Cu1—N5	2.0895 (19)	N9—C55	1.365 (3)
Cu1—N3	2.1013 (15)	N10—C59	1.136 (3)
Cu1—N2	2.1391 (16)	C31—C32	1.406 (3)
N1—C1	1.337 (2)	C31—C43	1.500 (3)
N1—C12	1.368 (2)	C32—C33	1.370 (3)
N2—C10	1.338 (2)	C32—H32	0.9300
N2—C11	1.370 (2)	C33—C34	1.409 (3)
N3—C15	1.341 (3)	C33—H33	0.9300
N3—C26	1.365 (2)	C34—C42	1.406 (3)
N4—C24	1.339 (3)	C34—C35	1.431 (3)
N4—C25	1.365 (2)	C35—C36	1.352 (3)
N5—C29	1.138 (3)	C35—H35	0.9300
C1—C2	1.413 (3)	C36—C37	1.435 (3)
C1—C13	1.491 (3)	C36—H36	0.9300
C2—C3	1.367 (3)	C37—C38	1.407 (3)
C2—H2	0.9300	C37—C41	1.408 (3)
C3—C4	1.411 (3)	C38—C39	1.367 (3)
C3—H3	0.9300	C38—H38	0.9300
C4—C12	1.406 (2)	C39—C40	1.409 (3)
C4—C5	1.432 (3)	C39—H39	0.9300
C5—C6	1.353 (3)	C40—C44	1.504 (3)
C5—H5	0.9300	C41—C42	1.431 (3)
C6—C7	1.433 (3)	C43—H43A	0.9600
C6—H6	0.9300	C43—H43B	0.9600
C7—C11	1.407 (2)	C43—H43C	0.9600
C7—C8	1.409 (3)	C44—H44A	0.9600
C8—C9	1.370 (3)	C44—H44B	0.9600
C8—H8	0.9300	C44—H44C	0.9600
C9—C10	1.413 (3)	C45—C46	1.412 (3)
C9—H9	0.9300	C45—C57	1.495 (3)
C10—C14	1.504 (3)	C46—C47	1.359 (3)
C11—C12	1.433 (3)	C46—H46	0.9300
C13—H13A	0.9600	C47—C48	1.402 (3)
C13—H13B	0.9600	C47—H47	0.9300
C13—H13C	0.9600	C48—C56	1.409 (3)
C14—H14A	0.9600	C48—C49	1.434 (3)
C14—H14B	0.9600	C49—C50	1.343 (3)
C14—H14C	0.9600	C49—H49	0.9300
C15—C16	1.412 (3)	C50—C51	1.432 (3)
C15—C27	1.498 (3)	C50—H50	0.9300
C16—C17	1.362 (3)	C51—C52	1.404 (3)
C16—H16	0.9300	C51—C55	1.406 (3)
C17—C18	1.395 (3)	C52—C53	1.358 (4)
C17—H17	0.9300	C52—H52	0.9300
C18—C26	1.415 (3)	C53—C54	1.417 (3)
C18—C19	1.431 (3)	C53—H53	0.9300
C19—C20	1.344 (4)	C54—C58	1.491 (3)
C19—H19	0.9300	C55—C56	1.433 (3)
C20—C21	1.442 (3)	C57—H57A	0.9600
C20—H20	0.9300	C57—H57B	0.9600
C21—C22	1.401 (3)	C57—H57C	0.9600
C21—C25	1.408 (3)	C58—H58A	0.9600
C22—C23	1.363 (3)	C58—H58B	0.9600
C22—H22	0.9300	C58—H58C	0.9600
C23—C24	1.414 (3)	C59—C60	1.453 (3)
C23—H23	0.9300	C60—H60A	0.9600
C24—C28	1.495 (3)	C60—H60B	0.9600
C25—C26	1.428 (3)	C60—H60C	0.9600
C27—H27A	0.9600	B1—F2	1.381 (3)
C27—H27B	0.9600	B1—F4	1.384 (3)
C27—H27C	0.9600	B1—F3	1.387 (3)
C28—H28A	0.9600	B1—F1	1.390 (2)
C28—H28B	0.9600	B2—F8	1.371 (3)
C28—H28C	0.9600	B2—F5	1.378 (3)
C29—C30	1.450 (3)	B2—F7	1.380 (3)
C30—H30A	0.9600	B2—F6	1.388 (3)
C30—H30B	0.9600	B3—F10	1.377 (3)
C30—H30C	0.9600	B3—F9	1.388 (3)
Cu2—N9	1.9931 (16)	B3—F11	1.390 (3)
Cu2—N7	1.9976 (16)	B3—F12	1.400 (3)
Cu2—N10	2.0894 (19)	B4—F15	1.359 (4)
Cu2—N6	2.1150 (18)	B4—F14	1.372 (4)
Cu2—N8	2.1295 (16)	B4—F13	1.386 (4)
N6—C31	1.339 (3)	B4—F16	1.391 (3)
N6—C42	1.375 (2)	B4A—F15A	1.364 (15)
N7—C40	1.337 (3)	B4A—F13A	1.391 (14)
N7—C41	1.371 (2)	B4A—F16A	1.392 (14)
N8—C45	1.339 (2)	B4A—F14A	1.392 (15)
			
N1—Cu1—N4	170.39 (6)	C41—N7—Cu2	112.88 (13)
N1—Cu1—N5	83.26 (6)	C45—N8—C56	118.32 (17)
N4—Cu1—N5	87.80 (7)	C45—N8—Cu2	132.63 (13)
N1—Cu1—N3	101.85 (6)	C56—N8—Cu2	109.03 (12)
N4—Cu1—N3	81.63 (6)	C54—N9—C55	119.16 (18)
N5—Cu1—N3	132.45 (7)	C54—N9—Cu2	126.84 (15)
N1—Cu1—N2	81.43 (6)	C55—N9—Cu2	113.58 (12)
N4—Cu1—N2	106.03 (6)	C59—N10—Cu2	164.90 (18)
N5—Cu1—N2	118.15 (7)	N6—C31—C32	121.4 (2)
N3—Cu1—N2	109.32 (6)	N6—C31—C43	119.0 (2)
C1—N1—C12	119.70 (15)	C32—C31—C43	119.6 (2)
C1—N1—Cu1	126.13 (13)	C33—C32—C31	120.5 (2)
C12—N1—Cu1	113.46 (12)	C33—C32—H32	119.8
C10—N2—C11	118.46 (16)	C31—C32—H32	119.8
C10—N2—Cu1	132.76 (12)	C32—C33—C34	119.6 (2)
C11—N2—Cu1	108.70 (12)	C32—C33—H33	120.2
C15—N3—C26	118.22 (16)	C34—C33—H33	120.2
C15—N3—Cu1	132.51 (13)	C42—C34—C33	116.9 (2)
C26—N3—Cu1	109.04 (12)	C42—C34—C35	119.5 (2)
C24—N4—C25	119.56 (16)	C33—C34—C35	123.6 (2)
C24—N4—Cu1	127.61 (13)	C36—C35—C34	121.2 (2)
C25—N4—Cu1	112.19 (12)	C36—C35—H35	119.4
C29—N5—Cu1	163.05 (18)	C34—C35—H35	119.4
N1—C1—C2	120.44 (17)	C35—C36—C37	120.8 (2)
N1—C1—C13	119.39 (16)	C35—C36—H36	119.6
C2—C1—C13	120.17 (17)	C37—C36—H36	119.6
C3—C2—C1	120.55 (18)	C38—C37—C41	117.60 (19)
C3—C2—H2	119.7	C38—C37—C36	123.49 (19)
C1—C2—H2	119.7	C41—C37—C36	118.90 (19)
C2—C3—C4	119.67 (17)	C39—C38—C37	119.55 (19)
C2—C3—H3	120.2	C39—C38—H38	120.2
C4—C3—H3	120.2	C37—C38—H38	120.2
C12—C4—C3	117.16 (17)	C38—C39—C40	120.4 (2)
C12—C4—C5	119.21 (17)	C38—C39—H39	119.8
C3—C4—C5	123.62 (17)	C40—C39—H39	119.8
C6—C5—C4	120.99 (17)	N7—C40—C39	121.12 (19)
C6—C5—H5	119.5	N7—C40—C44	119.46 (18)
C4—C5—H5	119.5	C39—C40—C44	119.42 (19)
C5—C6—C7	120.79 (18)	N7—C41—C37	122.17 (18)
C5—C6—H6	119.6	N7—C41—C42	117.45 (17)
C7—C6—H6	119.6	C37—C41—C42	120.36 (18)
C11—C7—C8	116.77 (17)	N6—C42—C34	123.25 (19)
C11—C7—C6	119.68 (17)	N6—C42—C41	117.48 (18)
C8—C7—C6	123.55 (17)	C34—C42—C41	119.24 (18)
C9—C8—C7	119.77 (18)	C31—C43—H43A	109.5
C9—C8—H8	120.1	C31—C43—H43B	109.5
C7—C8—H8	120.1	H43A—C43—H43B	109.5
C8—C9—C10	120.32 (18)	C31—C43—H43C	109.5
C8—C9—H9	119.8	H43A—C43—H43C	109.5
C10—C9—H9	119.8	H43B—C43—H43C	109.5
N2—C10—C9	121.24 (17)	C40—C44—H44A	109.5
N2—C10—C14	118.98 (17)	C40—C44—H44B	109.5
C9—C10—C14	119.76 (17)	H44A—C44—H44B	109.5
N2—C11—C7	123.42 (17)	C40—C44—H44C	109.5
N2—C11—C12	117.43 (16)	H44A—C44—H44C	109.5
C7—C11—C12	119.11 (16)	H44B—C44—H44C	109.5
N1—C12—C4	122.45 (16)	N8—C45—C46	121.50 (18)
N1—C12—C11	117.41 (15)	N8—C45—C57	119.98 (18)
C4—C12—C11	120.12 (16)	C46—C45—C57	118.51 (18)
C1—C13—H13A	109.5	C47—C46—C45	120.06 (19)
C1—C13—H13B	109.5	C47—C46—H46	120.0
H13A—C13—H13B	109.5	C45—C46—H46	120.0
C1—C13—H13C	109.5	C46—C47—C48	120.11 (19)
H13A—C13—H13C	109.5	C46—C47—H47	119.9
H13B—C13—H13C	109.5	C48—C47—H47	119.9
C10—C14—H14A	109.5	C47—C48—C56	117.08 (18)
C10—C14—H14B	109.5	C47—C48—C49	123.72 (19)
H14A—C14—H14B	109.5	C56—C48—C49	119.20 (19)
C10—C14—H14C	109.5	C50—C49—C48	120.8 (2)
H14A—C14—H14C	109.5	C50—C49—H49	119.6
H14B—C14—H14C	109.5	C48—C49—H49	119.6
N3—C15—C16	121.1 (2)	C49—C50—C51	121.59 (19)
N3—C15—C27	119.07 (18)	C49—C50—H50	119.2
C16—C15—C27	119.79 (19)	C51—C50—H50	119.2
C17—C16—C15	120.4 (2)	C52—C51—C55	117.0 (2)
C17—C16—H16	119.8	C52—C51—C50	124.0 (2)
C15—C16—H16	119.8	C55—C51—C50	118.93 (19)
C16—C17—C18	120.03 (19)	C53—C52—C51	119.7 (2)
C16—C17—H17	120.0	C53—C52—H52	120.1
C18—C17—H17	120.0	C51—C52—H52	120.1
C17—C18—C26	116.87 (19)	C52—C53—C54	121.0 (2)
C17—C18—C19	123.94 (19)	C52—C53—H53	119.5
C26—C18—C19	119.2 (2)	C54—C53—H53	119.5
C20—C19—C18	120.98 (19)	N9—C54—C53	120.2 (2)
C20—C19—H19	119.5	N9—C54—C58	119.1 (2)
C18—C19—H19	119.5	C53—C54—C58	120.7 (2)
C19—C20—C21	121.33 (19)	N9—C55—C51	122.96 (18)
C19—C20—H20	119.3	N9—C55—C56	117.21 (16)
C21—C20—H20	119.3	C51—C55—C56	119.76 (18)
C22—C21—C25	117.11 (19)	N8—C56—C48	122.85 (17)
C22—C21—C20	123.96 (19)	N8—C56—C55	117.45 (17)
C25—C21—C20	118.9 (2)	C48—C56—C55	119.64 (17)
C23—C22—C21	119.65 (19)	C45—C57—H57A	109.5
C23—C22—H22	120.2	C45—C57—H57B	109.5
C21—C22—H22	120.2	H57A—C57—H57B	109.5
C22—C23—C24	121.1 (2)	C45—C57—H57C	109.5
C22—C23—H23	119.5	H57A—C57—H57C	109.5
C24—C23—H23	119.5	H57B—C57—H57C	109.5
N4—C24—C23	119.89 (19)	C54—C58—H58A	109.5
N4—C24—C28	119.77 (18)	C54—C58—H58B	109.5
C23—C24—C28	120.33 (19)	H58A—C58—H58B	109.5
N4—C25—C21	122.67 (18)	C54—C58—H58C	109.5
N4—C25—C26	117.53 (16)	H58A—C58—H58C	109.5
C21—C25—C26	119.77 (17)	H58B—C58—H58C	109.5
N3—C26—C18	123.20 (18)	N10—C59—C60	179.8 (3)
N3—C26—C25	116.92 (16)	C59—C60—H60A	109.5
C18—C26—C25	119.80 (18)	C59—C60—H60B	109.5
C15—C27—H27A	109.5	H60A—C60—H60B	109.5
C15—C27—H27B	109.5	C59—C60—H60C	109.5
H27A—C27—H27B	109.5	H60A—C60—H60C	109.5
C15—C27—H27C	109.5	H60B—C60—H60C	109.5
H27A—C27—H27C	109.5	F2—B1—F4	107.83 (18)
H27B—C27—H27C	109.5	F2—B1—F3	108.17 (19)
C24—C28—H28A	109.5	F4—B1—F3	110.45 (19)
C24—C28—H28B	109.5	F2—B1—F1	109.42 (18)
H28A—C28—H28B	109.5	F4—B1—F1	111.20 (18)
C24—C28—H28C	109.5	F3—B1—F1	109.69 (17)
H28A—C28—H28C	109.5	F8—B2—F5	111.0 (2)
H28B—C28—H28C	109.5	F8—B2—F7	107.5 (2)
N5—C29—C30	178.7 (3)	F5—B2—F7	106.3 (2)
C29—C30—H30A	109.5	F8—B2—F6	112.6 (2)
C29—C30—H30B	109.5	F5—B2—F6	110.27 (19)
H30A—C30—H30B	109.5	F7—B2—F6	108.77 (19)
C29—C30—H30C	109.5	F10—B3—F9	111.1 (2)
H30A—C30—H30C	109.5	F10—B3—F11	110.6 (2)
H30B—C30—H30C	109.5	F9—B3—F11	109.4 (2)
N9—Cu2—N7	171.49 (7)	F10—B3—F12	109.1 (2)
N9—Cu2—N10	84.78 (7)	F9—B3—F12	108.6 (2)
N7—Cu2—N10	86.72 (7)	F11—B3—F12	107.9 (2)
N9—Cu2—N6	103.17 (7)	F15—B4—F14	110.4 (3)
N7—Cu2—N6	81.92 (6)	F15—B4—F13	108.9 (3)
N10—Cu2—N6	128.15 (7)	F14—B4—F13	109.7 (2)
N9—Cu2—N8	81.35 (6)	F15—B4—F16	107.8 (3)
N7—Cu2—N8	103.69 (6)	F14—B4—F16	109.4 (3)
N10—Cu2—N8	123.42 (7)	F13—B4—F16	110.6 (2)
N6—Cu2—N8	108.43 (6)	F15A—B4A—F13A	105.9 (13)
C31—N6—C42	118.24 (18)	F15A—B4A—F16A	108.9 (13)
C31—N6—Cu2	132.65 (14)	F13A—B4A—F16A	112.0 (14)
C42—N6—Cu2	108.99 (13)	F15A—B4A—F14A	115.4 (15)
C40—N7—C41	119.19 (17)	F13A—B4A—F14A	106.1 (12)
C40—N7—Cu2	127.65 (14)	F16A—B4A—F14A	108.5 (13)
